# Public health evolutionary biology of antimicrobial resistance: priorities for intervention

**DOI:** 10.1111/eva.12235

**Published:** 2014-12-11

**Authors:** Fernando Baquero, Val F Lanza, Rafael Cantón, Teresa M Coque

**Affiliations:** 1 Departamento de Microbiología, Hospital Universitario Ramón y Cajal, Instituto Ramón y Cajal de Investigación Sanitaria (IRYCIS)Madrid, Spain; 2 Unidad de Resistencia a Antibióticos y Virulencia Bacteriana asociada al Consejo Superior de Investigaciones Científicas (CSIC)Madrid, Spain; 3 CIBER Epidemiología y Salud Pública (CIBERESP)Madrid, Spain; 4 Spanish Network for the Research in Infectious Diseases (REIPI RD12/0015), Instituto de Salud Carlos IIIMadrid, Spain

**Keywords:** antibiotic resistance, complex causes, complex interventions, emergence of antibiotic resistance, invasion by antibiotic resistance, occupation by antibiotic resistance, public health evolutionary biology

## Abstract

The three main processes shaping the evolutionary ecology of antibiotic resistance (AbR) involve the emergence, invasion and occupation by antibiotic-resistant genes of significant environments for human health. The process of emergence in complex bacterial populations is a high-frequency, continuous swarming of ephemeral combinatory genetic and epigenetic explorations inside cells and among cells, populations and communities, expanding in different environments (migration), creating the stochastic variation required for evolutionary progress. Invasion refers to the process by which AbR significantly increases in frequency in a given (invaded) environment, led by external invaders local multiplication and spread, or by endogenous conversion. Conversion occurs because of the spread of AbR genes from an exogenous resistant clone into an established (endogenous) bacterial clone(s) colonizing the environment; and/or because of dissemination of particular resistant genetic variants that emerged within an endogenous clonal population. Occupation of a given environment by a resistant variant means a permanent establishment of this organism in this environment, even in the absence of antibiotic selection. Specific interventions on emergence influence invasion, those acting on invasion also influence occupation and interventions on occupation determine emergence. Such interventions should be simultaneously applied, as they are not simple solutions to the complex problem of AbR.

## Public health evolutionary biology

The incorporation of evolutionary thinking is essential for Medicine and Public Health to achieve its full potential (Nesse et al. 2010; Stearns 2012; Losos et al. 2013). Evolutionary Medicine is a promising new and fertile field for research on processes producing harm to humans, animals and possibly in the health of the biosphere (Shanks and Pyles 2007; Stearns and Koella 2007; Trevathan 2007; Gluckman et al. 2009; Nesse et al. 2010, 2012). Note that Medicine itself is evolving from the science of patient care to a science that strives to promote and preserve health, not only human and animal, but also global health: the health of the biosphere (Baquero 2009; Baquero et al. 2010; Rubin et al. 2013). Public Health has a long tradition of considering the environmental causes of illnesses and has been directed to exploring the processes leading to the deterioration of health in mankind. In fact, the word ‘epidemiology’ means ‘what is upon the people’, in other words, the consequences of processes starting in the environment that cause deleterious effects on human populations. Evolution is a science of processes, trying to analyse the consequences of the interaction between biological entities and the environment, in a way that these entities might be modified in quantity or quality, changes that could result in the modification of their healthy relations (Linnan and Steckler 2002; Baquero et al. 2010; Rayner and Lang 2013; Friis and Sellers 2014). Antibiotic resistance (AbR) is a pathological effect of an altered human–microbes interaction mostly due to *processes* resulting from anthropogenic alterations in different environments, with consequences in human health and possibly in the health of the biosphere. In evolutionary theory, processes are the generators of patterns (the observable status of any ordered reality), and responsible for the emergence, variation and maintenance of novel patterns (Chapleau and Johansen 1988). Consequently, the analysis and control of AbR require eco-evolutionary thinking in a Public Health dimension, that is the development of a Public Health evolutionary biology of AbR, leading to evolutionary applications (Nesse et al. 2010; Baquero et al. 2011). In this article, we review the main processes leading to the current abnormal patterns of AbR, to develop and adopt intervention measures directed to limit its negative consequences on Public Health.

## The evolution of antimicrobial resistance

The possibility of studying evolution ‘in real time’ is one of the highest ambitions of biological sciences. That might be experimentally accomplished in small fishes (goopies) submitted to predator-prey evolution (Endler 1980; Reznick et al. 1990). However, AbR is the best possible example of evolution of biological entities acting ‘in real time’ (De la Cruz and Davies 2000; Rosenthal and Elowitz 2012; Toprak et al. 2013). Interestingly, AbR can be considered a collective compensatory bacterial action tending to restore a pristine status of equilibrium between colonized hosts and environments (Brown et al. 2012; Viana et al. 2014). This action shows the robustness of the complex associations of bacteria and their ecological niches, resisting to alterations resulting from the anthropogenic production and release in the environment of massive quantities of antimicrobials (Kümmerer and Henninger 2003; Knapp et al. 2010, 2011; Gillings and Stokes 2012; Gillings 2013). Natural antimicrobial agents are probably more ‘signals’ than ‘weapons’ (Linares et al. 2006). Intermicrobial signalling agents exert their function at extremely tiny concentrations, so that the exposure to huge concentrations of signals has deleterious effects not only on bacterial cells, but also on the entire wiring system of microbial ecosystems. AbR might be the reaction to such an anthropogenic action, and therefore, we, humans, are a main triggering agent of the problem; microbes only try to assure the permanence of their healthy links with the environment. Of course, AbR genes exist since bacteria emerged, but we enhanced their concentration proportionally to the release of antibiotics in certain ecological niches (Rolain et al. 2012; Baquero et al. 2013). ‘New paradigms’ in antibiotic use have been suggested to reduce these effects during treatment of infections, including narrow spectrum, more pathogen directed-drugs, and emphasizing actions to control host-response (Casadevall 1996; Clatworthy et al. 2007; Masterton 2009; Alemayehu et al. 2012; Rolain et al. 2012).

The use of industrial antibiotics has significantly contributed to human health, facilitating the progress of modern medicine, but simultaneously, we might have provoked the contrary effect. In this ‘Antibiotic Paradox’ (Levy 2002), the immediate beneficial impact on health occurs at the expenses of severe, perhaps irreversible effects on bacterial populations (Rolain et al. 2012; Baquero et al. 2013), finally resulting in novel threats for global health. To a certain extent, the success of AbR has re-equilibrated the deep environmental damage produced by antimicrobial agents on the microbiosphere, but the equilibrium is a ‘new equilibrium’ with novel compositions of bacterial communities, eventually comprising a massive number of genetic elements (as integrons, transposons, plasmids and phages) able to efficiently acquire and spread AbR genes which now pollute the entire biosphere (Costello et al. 2012; Gillings 2013; Losos et al. 2013). As example, it has been estimated that, resulting from anthropogenic activities, 10^19^ bacteria containing class 1 integrons (a sophisticated genetic platform able to recruit AbR genes) are released in sewage in the United Kingdom every year, high integron concentration also being detected in different environmental settings and hosts (Gaze et al. 2011; Stalder et al. 2012). Similarly, the number of bacteriophages increases close to human environments (Muniesa et al. 2013). The acquisition of new traits, or novel combinations of traits able to confer new adaptive phenotypes may result in the emergence of new evolutionary lineages (defined as *evolutionary novelty*) (Pigliucci 2008). In some cases, such ‘novelty’ implies profound changes as a result of dynamics of mutation, gene flow, drift and selection that led to ‘hopeful monsters’. This classical and highly controversial concept coined by Goldschmidt in early 20th century (Goldschmidt 1933; Dittrich-Reed and Fitzpatrick 2013) emphasizes the stochasticity of their appearance, and, to a certain extent, its unpredictable nature (Baquero and Tobes 2013; Chen et al. 2014; Croucher et al. 2014).

How has AbR reached a planetary spread among all kind of environments? We cannot discard that other bacterial adaptive traits may also be spreading with high efficiency among bacterial populations and that AbR provides more easy-to-detect phenotypes of obvious importance in Public Health. In any case, it seems reasonable to think that humans have – through the excessive use of antibiotic, biocides and industrial pollution – accelerated the building-up and selection of genes and genetic platforms involved in AbR. General factors derived from societal changes in human populations and the environment, including changes in land use (intensified human encroachment on natural environments), and globalization of planet biology – including human population growth; livestock and production methods; international travel or long distant trade of humans, animals and vegetables, breakdown in public health infrastructure, and eventually geo-anthropological changes (such as global warming), might have also contributed to the Earth invasion by AbR bacteria (Smolinski et al. 2003; Baquero 2009; Karesh et al. 2012; Wellington et al. 2013). The rising consciousness about this multicausal complexity requires a novel reconsideration of the priorities among possible interventions aiming to fight AbR (Ashbolt et al. 2013; Bush et al. 2011; Petticrew 2011; Costello et al. 2012; Moore et al. 2014; Viana et al. 2014). In fact, these priorities are dependent of our current situation, and in particular if we have trespassed (locally or globally) the nonreturn red line of AbR.

## Evolutionary processes in AbR: approaching a nonreturn red line

The three main processes involved in the evolutionary ecology of AbR are as follows: first, the emergence; second, the invasion; and third, the occupation by AbR genes of significant environments for human health. Single approach interventions to influence each one of these processes are quite different but often fail. That is due to the overwhelming lack of knowledge in the AbR genes reservoir dynamics and its consequences on human health, precluding a risk assessment analysis (McEwen 2012; Ashbolt et al. 2013; Da Costa et al. 2013; Rizzo et al. 2013; Viana et al. 2014). Until recently, most of the interventions have been focused only on the prevention of the emergence of resistance at patients level (for instance with a personalized pharmacokinetic and pharmacodynamic use of antibiotics, strategies based on antibiotic mixing or cycling, preventive oral or intestinal decontamination, or probiotic use), and in the prevention of invasion of particular compartments, as hospitals or farms (by regulating the proper consumption of antibiotics to reduce selection, and also by isolation or containment measures of infected and colonized hosts) (Kollef 2006; Baquero 2007; De Regt et al. 2010; Daneman et al. 2013).

Reaching the third process, occupation, a high-risk nonreturn red line, is crossed. Beyond this line, the selection, spread and fixation of AbR genes, platforms and organisms in human-significant environments no longer depend on the clinical use of antibiotic agents. A good example is the *bla*carbapenemase genes which, once are established in multi-environment pathogens acquired an overwhelming ‘*betweenness*’ among a large network of evolutionary elements, favoured by rapid spreading of mobile genetic elements (MGEs) and high-risk bacterial clones (Djordjevic et al. 2013; Gillings 2013). Consequently, the influence on the process of interventions based on conventional restrictions of antibiotic use or hosts’ isolation procedures might not be successful beyond the red line, forcing us to explore the possibilities of other type of interventions (Baquero et al. 2011; Laxminarayan et al. 2013; Fowler et al. 2014) necessarily applied within the frame of global ecological improvements.

## The process of emergence of AbR

### Emergence

In the epidemiological and Public Health perspective, the term ‘emergence’ intuitively indicates the act of becoming known or coming into view. In evolutionary theory, ‘emergence’ is a key term that has widely been used in disparate scientific disciplines under very diverse perspectives, with major applications in social systems, evolution of ecosystems and co-evolution (Goldstein 1999; Corning 2002; De Haan 2006). The term, firstly coined in late 19th century to fill some philosophical gaps in evolutionary theory, was reanalysed under the light of the complexity theory in the 20th century. This shift in the scientific paradigm, now accelerated by the introduction of systems biology (Mazzocchi 2008; Fang and Casadevall 2011), resulted in different concepts and types of emergence (De Haan 2006; Alexander et al. 2012). However, in all of them, the perception of emergence requires *growth* (Alexander et al. 2012), that is, something might exist, but only ‘emerges’ if it reaches the abundance to hit the boundaries of visibility. In this sense, phenotypic visibility depends on the potency of our instruments, the amplitude of our goals and the accepted criteria for defining the phenomena that we wanted to observe.

For instance, we should consider different definitions of AbR (Davison et al. 2000; Simjee et al. 2008; Martinez and Silley 2010). From a clinical point of view, AbR emerges when unexpected failures in antibiotic therapy occur in some patients; for epidemiologists and Public Health officers, when AbR becomes sufficiently frequent to be considered a collective problem; for field microbiologists, when a novel phenotype of resistance is detected; for molecular microbiologists, when a previously unknown ‘antibiotic-resistant’ gene appears in a metagenomic sample or is characterized in a ‘resistant bacteria’. It is not difficult to predict than when a resistance mechanism reaches the visibility level at hospitals and stockyards, this mechanism is already certainly well spread (has experimented ‘growth’) among bacterial populations in the community (emergence depends on *growth*).

The term ‘emergence’ is a property of complex systems and therefore applies to the complex biological entities. In that sense, ‘emergence’ means the emergence of a complex singular pattern (system) from relatively simple interactions among pieces (Baquero 2004). In AbR, ‘emergence’ derives from: (i) spontaneous local mutational variation and the functional interaction of such variation with the epistatic interactive network inside the organism; the immediate consequences of such emergence in Public Health are hard to assess as frequently results in ‘low level’ resistance, without consequences in the categorization of bacteria as ‘resistant’; and (ii) more frequently, from a cumulative combinatory interplay of mutations, genes, gene-capturing platforms, genetic vehicles integrating these platforms and spreading among bacterial clones, bacterial populations and complex communities, as the microbiota. This emergence generally involves ‘high-level expression’ phenotypes and consequently better detected in clinical microbiology laboratories; in this sense, such ‘emergence’ can be directly recognized as a bacterial adaptive response to the selective pressure exercised by antibiotics.

### Emergence and combinatorial processes

Microbial complex systems arrange their hierarchical components into adaptive structures with emergent properties. Such structures are able to create new strategies and modify existing ones to adapt to changing environmental conditions (Coffey 1998). Antibiotics are a major source of micro-environmental changes. Antibiotics also influence a wide diversity of processes including the increase in the frequency of diverse lateral gene transfer (LGT) as conjugation, transduction and recombination (Baquero et al. 2013; Gillings 2013; Modi et al. 2013). These processes facilitate the penetration of either widespread genetic elements to new bacterial hosts or novel genetic elements to widespread hosts, the creation of hotspots that favours the assembly of complex mosaic structures, and the generation of complex arrangements able to provide emergent properties and increase the complexity of the host and the system (Goldschmidt 1933; Partridge 2011; Toleman and Walsh 2011; Chen et al. 2014; Croucher et al. 2014). Other human activities mentioned above also contribute to select for multi-environment opportunistic pathogens with high transmissibility and propensity for LGT, thus amplifying the chances to arise emerging properties located either on MGEs or on chromosome (Dethlefsen et al. 2007, 2008; Gillings and Stokes 2012; Looft and Allen 2012; Petty et al. 2014). In fact, each one of the interactions between these elements should be to a certain extent synergistic. Synergistic effects of various kinds have overall played a major causal role in the evolutionary process and in the evolution of cooperation and complexity in particular evolutionary systems (Corning 2002; Livnat 2013). In AbR, the success (leading to the ‘emergence’ in Public Health optics) of a particular resistant bacterial clone might depend on the success of a particular resistance mutation in an optimal epigenetic context (Rosenthal and Elowitz 2012; Livnat 2013; Martínez 2013; Toprak et al. 2013), and its location on a successful genetic background (see below). The history of most commonly identified AbR genes follows its original capture by different genetic units and further mobilization of novel ‘operational units’ containing such AbR genes into composite platforms (often comprising integrons, transposable elements, and/or insertion sequences) within plasmids that facilitate multiple DNA rearrangements among disparate genetic entities. As examples, promiscuous FII chimeric plasmids widely spread among Enterobacteriaceae, which are responsible for the current pandemic spread of *bla*_CTX-M-15_, or *bla*_TEM,_ among other AbR genes (Baquero 2004; Partridge and Hall 2004; Partridge et al. 2011; Partridge and Iredell 2012). These plasmids harbour operational genetic platforms containing gene capture units as IS*ECp1*, IS*26* or IS*CRs*, thus recruiting diverse AbR genes (Partridge 2011; Partridge et al. 2011; Toleman and Walsh 2011) In many cases, the final success of resistant clones depends on the acquisition of adaptive features unrelated to resistance, facilitating in some of them to spread between hosts and/or environments (epidemicity) (Jackson et al. 2011; Baquero and Tobes 2013; Chen et al. 2014; Croucher et al. 2014; Petty et al. 2014).

### Emergence, gradients and environmental heterogeneity

On all grounds, possibly a faithful image of the basic process of ‘emergence’ in complex bacterial populations is a high-frequency, continuous swarming of ephemeral combinatory genetic and epigenetic explorations inside cells and among cells, inside and among bacterial populations and communities expanding in different environments (migration) creating the dense stochastic variation required for evolutionary progresses. As in the case of mutational events, bacteria continuously play a risky game, exposing themselves to decreased fitness, to feed a ‘capital of diversity’ and consequently the possibility to adapt to unexpected deleterious events. This is an example of *allostasis*, maintaining stability through change, a fundamental process through which organisms actively adjust to both predictable and unpredictable events (McEwen and Wingfield 2003; Holt 2004; Perron et al. 2007; Hermsen and Hwa 2010). Unfortunately, if from one side, the ‘mutation rate’ of microorganisms is considered relatively constant and is in fact known for many microorganisms, these ‘combinatorial rates’ are extremely difficult to establish, as they depend on many population and environmental parameters (Hermsen et al. 2012; Martínez and Baquero 2014). As in the case of mutational variation, many combinatorial products are probably neutral in stable environments and might eventually spread only driven by random genetic drift (Swithers et al. 2012; Król et al. 2013). If neutral evolution is frequently acting in emergent patterns, as in the case of mutational variation or migration, both the emergence and the advantage of certain combinations are emphasized by the bacterial exposure to variable environments. Such environmental variation also results from bacterial action (including colonization and pathogenesis) and, most importantly, depends on the physical structure of the environments. One of the major reasons for environmental heterogeneity is gradient formation.

Gradients are the consequence of the diffusion of chemical and physical conditions in a continuous space. Gradients are generated by the asymmetrical presence of such conditions, which are concentrated at certain points of the space. When antibiotics are given to a patient (or to livestock individuals), there is a wide set of gradients of antibiotic concentrations in the organic spaces, so that bacteria are confronted with a diversity of concentrations (Baquero and Negri 1997). Also, bacterial growth itself provokes gradients, such as gradients of nutrients or catabolites, or gradients in acidity, or amensalistic substances. Of course, a complex array of intergradient interactions is expected to occur in nature, leading to multidimensional complex gradients. The important message is that every spatial bit of a gradient might serve as a ‘selection point’ allowing the local enrichment of a particular ‘emergent’ combinatory product arising in bacteria (Baquero and Negri 1997). For instance, gradients of antibiotic concentrations allow the selection of different bacterial mutants in different gradient levels (Negri et al. 2000). Evading competition in such a way (Hermsen et al. 2012), concentration gradients create a huge ‘environmental spatial diversity’, which is confronted with the diversity of combinatorial biological variants, allowing the discrete selection of combinations with even small phenotypic differences and allowing step-by-step evolution (from low-level to high-level resistance) (Baquero et al. 1998; Greulich et al. 2012; Hermsen et al. 2012). Using such a stair-case process, gradients facilitate the rate of AbR evolution (Zhang et al. 2011; Hermsen et al. 2012). This model of adaptation is greatly dependent on bacterial growth (Deris et al. 2013), the type of antibiotic (Novais et al. 2010; Toprak et al. 2013; Händel et al. 2014) and therapeutic regimens as a whole (Perron et al. 2006) and maybe other host factors (Reeves et al. 2011).

In summary, the ‘emergence’ of AbR depends on the complex formation of successful combinations of biological traits and elements that result from stochastic (unpredictable) interactions involving processes of mutation, conjugation, recombination (modularization), migration, growth and death. The possibility of these interactions will certainly depend on a number of parameters, including the frequency of each one of the biological elements and the ecological connectivity between them (Popa et al. 2011; Tamminen et al. 2012; Toussaint and Chandler 2012; Livnat 2013; Boon et al. 2014; Martínez and Baquero 2014). For each one of these emergent patterns, there is a chance of an initial local penetration in heterogeneous complex (mosaic) environments offering a wealth of selective points produced by gradients.

## The process of invasion by AbR

It is not always easy to differentiate emergence from invasion, as emergence should be immediately followed by the local spread of the emergent structure, a first ‘mini-invasion’ (Fraser et al. 2005). Many emergent and potentially dreadful combinations for the spread of AbR are probably lost after its stochastic birth. Those associated with more successful phenotypes become fixed and start the invasive process, depending on the local presence of a selective condition, either the presence of antibiotics, or other environmental factors (Livnat 2013; Boon et al. 2014). Invasion refers to the process by which AbR significantly increases in frequency in a given (invaded) environment. It might result from different events, led by external invaders, or by conversion to resistance of the resident populations (Levin et al. 1988). That is summarized below.

### Exogenous invasion

Exogenous invasion led to the local multiplication and spread of particular resistant exogenous species or clone, which displaces other susceptible clones of the same or different species; thus implying local clonal shifts. It is to note that exogenous invasion by a resistant clone might be favoured, but do not necessarily require antibiotic selection. The success of the invading organism might be explained by many other reasons, mostly related with adequacy of its metabolic features, local colonization-transmission abilities, derived from genotype and phenotypic diversity (Buckee et al. 2008; Ajao et al. 2011; Heithoff et al. 2012; Ng et al. 2013; Forsman 2014; Meador et al. 2014). In exogenous invasion, the process is led by environment-invasive clones that carry AbR determinants.

The mechanism(s) by which particular invasive alien resistant clones are able to successfully invade a given compartment (as the intestinal tract) depends on the clones themselves and the recipient environment. Bacterial clones differ in their invasion ability, particularly if the initial invading population is of low bacterial density. Organisms with small *propagulum* (the number of cells required for colonization) will definitely require excellent adhesive properties to compensate the natural cellular turnover of the intestinal tract, or, eventually, be very efficient in local multiplication, taking advantage of the available local nutrients (nutrient-niche hypothesis) (Maltby et al. 2013; Meador et al. 2014). In general, novel resistant clones will need to adapt and survive in the novel environment, and hypermutable organisms could have an advantage for such adaptation (Baquero et al. 2004; Perron et al. 2010; Wang et al. 2013) resulting in the selection of variants with large-effect mutations over those brother variants of small effect (clonal interference) (Barroso-Batista et al. 2014). Similarly, clonal interferences leading to differences in temporal or spatial clonal prevalence (local ‘invasions’) also result from plasmid adaptive dynamics in the populations (Hughes et al. 2012; Freitas et al. 2013). It is to be noticed that intestinal colonization serves as a strong selective bottleneck for the best exogenous colonizers, which might explain the frequent high efficiency of transmission during early stages of epidemics, including invisible ‘epidemics’ of commensal resistant organisms.

The recipient environment might be variable in the acceptation of foreign invaders, determined by various favouring conditions of the host, for instance, the nutritional and immunological landscape of the intestine (Buckee et al. 2008; Ng et al. 2013; Yoon et al. 2013), and complex ‘colonization-resistance’ profiles of the local microbiota (He et al. 2010; Buffie and Pamer 2013). The invasion of exogenous resistant clones will be facilitated by a ‘common nutritional-immunological landscape’ of a community of individuals living under the same circumstances, as the ‘best invaders’ for a given individual are probably the same as for other individuals in the group. Of course, the exposure of antimicrobial agents contributes to the invasive process of resistant organisms, eventually decreasing ‘colonization-resistance’. All these factors often coincide in groups of individuals in farms, hospitals or crowded groups under poor sanitation facilities, which frequently plays an important role in the process of local group-invasion of AbR.

### Endogenous conversion

Endogenous conversion occurs: (i) because of the spread of AbR genes from an exogenous resistant clone into well-established, high-density bacterial clone(s) colonizing the environment (endogenous); and/or (ii) because of dissemination of particular resistant genetic variants that has emerged within an endogenous colonizing clonal population. In endogenous conversion, clonal shifts might be weak, and the invasive process depends on the local spread of invasive genes and genetic variants, following selection, horizontal gene transfer or recombination.

The term ‘conversion’ means that something already existing in a particular environment acquires an adaptive trait present only in part of analogous entities coexisting in the same setting. This term has been used to illustrate the unidirectional transfer of genetic ‘variant’ material from a ‘donor’ sequence to a highly homologous ‘naïve acceptor’ sequence following recombination (Santoyo and Romero 2005; Hao and Palmer 2012). There are frequent examples of gene conversion in AbR, as in the case of *rrl* chromosomal genes, which are progressively ‘converted to linezolid resistance, the resistance of *Mycobacterium smegmatis* to aminoglycosides and the resistance of *Streptococcus pneumoniae* to evernimicin (Prammananan et al. 1999; Adrian et al. 2000; Boumghar-Bourtchaï et al. 2009)**.** Conversion facilitates acquisition of the advantageous trait by kin sequences or kin organisms, minimizing further changes.

Horizontal gene transfer of AbR, and other adaptive traits, is facilitated by phylogenetic neighbourhood (Wiedenbeck and Cohan 2011; Fernandez-Lopez et al. 2014) and also by sharing the same ecological niches as in the ecological architecture of the microbiome (Smillie et al. 2011). In both cases, horizontal gene transfer favours the functional integrity of bacterial consortia, particularly in compartmentalized environments with multidimensional genotypic spaces and many local optima (Jain 2007), minimizing bacterial competition between clones with different mutations (Perron et al. 2012b). The integrity of multistratified (shell model) bacterial groups with phylogenetic- and functional kin-relationships (such as different coexisting clones of the same species) favours collective adaptations, assuring the robustness of microbial associations when facing stressful conditions, such as antibiotic exposure, and, in general, environmental uncertainty (Yi and Dean 2013). The local multiclonal species structure in spatially structured environments is based on trade-offs between competitive-adaptive dispersal activities (Lin et al. 2013). Local spread of AbR is highly facilitated by such ‘never at equilibrium’ multiclonal intraspecific structure, explaining why the invasion of an antibiotic-resistant clone is frequently followed by an unexpected burst of other (coexisting) clones harbouring the same resistant trait, eventually giving rise to allodemic situations, where resistance is spread in a bunch of organisms, not as a single organism ‘epidemics’ (Baquero et al. 2002).

## The process of occupation by AbR

Occupation of a given environment by a resistant variant means a permanent establishment of this resistant organism in this environment, even in the absence of specific antibiotic selection. At the level of community ecology, this concept corresponds to that of ‘fixation’ of particular genotypes that emerged by mutational and or recombinatorial events in a population. Of course, this notion of ‘occupation’ can be downloaded to the gene level, when a resistance gene is permanently maintained in a particular environment, either because of being harboured by an occupant organism (Petty et al. 2014) or because of its frequent horizontal transfer among local bacterial communities (Stokes and Gillings 2011; Partridge and Iredell 2012; Berrazeg et al. 2014).

The easiest way of achieving occupation is the localization of AbR genes in well-adapted clones colonizing humans or animals or in well-adapted transferable genetic elements. When a AbR gene conferring a relevant phenotype enters a highly connected colonizer, the risk of permanence of AbR in the host population or environment is significantly increased (Beiko et al. 2005; Popa et al. 2011; Tamminen et al. 2012; Toussaint and Chandler 2012). These clones are linked to their hosts (including the host microbiota) by a dense network of interactions, from metabolic to immunological ones (Yoon et al. 2013). Such clones are easily spreading among kin-populations, as from mother to child, in the so-called vertical transmission of AbR (Baquero and Nombela 2012; Kothari et al. 2013), or among environments with high-density populations as homes, farms and health care centres irrespectively (but eventually favoured) from antibiotic exposure (Valverde et al. 2008; Stokes and Gillings 2011; Djordjevic et al. 2013). Moreover, these clones frequently harbour well-adapted plasmids maintained by selection-transfer balance, which might disseminate AbR among genetic exchange communities, even in the absence of antibiotic exposure, serving as raw platforms for the evolution of novel beneficial functions (Boon et al. 2014; Knöppel et al. 2014). In other cases, AbR genes are recruited by highly connected capture units (e.g. integrases) from which the segregation is rare and hence, the fixation is expected to occur under favourable conditions (Garriss et al. 2009; Partridge 2011; Toleman and Walsh 2011; Partridge and Iredell 2012). Interestingly, there seems to be frequent limits for full replacement of antibiotic susceptible for resistant populations in most environments, including the intestinal microbiota. The preservation of susceptible populations, and their coexistence in equilibrium with resistant ones, is mainly based on the existence of two surviving strategies: that of ‘producer organisms’, breaking down antibiotics and the one of ‘cheaters’, living on the edges of the producers (Baquero et al. 1985; Dugatkin et al. 2005, 2008). The more important the occupation by resistant organisms, more important is local detoxification of antibiotics, resulting in protection of susceptible ones. In target-mutant-resistant organisms, not detoxifying antibiotics, the maintenance of susceptible populations might be due to the cost of resistance (Björkman et al. 2000), most importantly if influencing colonization abilities.

Occupancy of supra-individual spaces, as hospitals, long-term facilities, families and household and farms, is facilitated by cross-transmission of the resistant organisms between colonized individuals, either by person-to-person contact (eventually including health workers) or/and involving contaminated equipment, water and food; indeed, antibiotic exposure facilitates occupancy (Valverde et al. 2008; Stokes and Gillings 2011; Djordjevic et al. 2013). Note that in these complex environments, bacterial clonal populations are submitted to oscillatory events by largely unknown, certainly multifactorial, reasons.

Occupation by resistance genes and resistant bacterial species in geographically defined human populations has been recently illustrated by metagenomic studies in intestinal microbiomes (Forslund et al. 2013, 2014). The higher gut microbiome ‘resistance capacities’, considering the density of ‘resistance genes’ to different antibiotics and in various species, corresponds to geographic contexts where both medical and food production antibiotic use are very high, suggesting a frequent circulation of resistance among humans and human-connected ecosystems, including food animals (Stokes and Gillings 2011; Djordjevic et al. 2013; Gaze et al. 2013; Szmolka and Nagy 2013). Indeed, the need for ‘global solutions’ addressing also the differences between local ‘microbial markets’ has been recently stressed (Jamison et al. 2013; Werner et al. 2014).

## Interventions against AbR

Interventions are expected to target the processes of emergence, invasion and occupation of AbR in particular environments. However, as shown in the former paragraphs, these processes are the product of a tangled web of causal influences, that is have a complex, nonlinear causation. Moreover, complex causation is often a weak causation. Of course, a solid causality principle is based on the observation that something happening regularly will always happen under similar circumstances, but that requires complete information of the situation while the causal frame of AbRs still contains many uncertainties.

Recent programmes recognize the need to establish complex medical interventions in areas of prevention (social, behavioural, environmental), diagnostics and therapy, with emphasis on personalized medicine and genomic medicine (Issa et al. 2014).

### Complex causation and complex interventions

In accordance with a classical declaration of the Medical Research Council's evaluation framework, complexity influences interventions not only because of the number of interacting components, but also because of the number and difficulty of behaviours required by those delivering or receiving the intervention (Charani et al. 2014); the number of groups or organizational levels targeted by the intervention (Grundmann 2014; Tomson and Vlad 2014; Viana et al. 2014) the number and variability of outcomes; and the degree of flexibility or tailoring of the intervention permitted (Craig et al. 2008; Petticrew 2011; Viana et al. 2014).

In AbR, a number of possible interventions have an apparent simplicity, but in fact, they influence complex medical and evolutionary landscapes, that is they might result in many other effects, variable in different places and some of them eventually unwanted. For instance, the intervention ‘antibiotic restriction in use to decrease AbR’ might increase morbidity or mortality of infectious diseases in some areas or situations (Meyer et al. 2009); the intervention ‘use combination antibiotic therapy to decrease the risk of emergence of resistance’ might result in the increase of multiresistant strains (Perron et al. 2012a); or the intervention ‘vaccination against resistant clones to decrease AbR’ might eliminate well host-adapted organisms and force its replacement by other (more virulent) bacteria. Note that in all these examples, the ‘interventions’ could in fact reduce AbR, but might also lead to biological changes, eventually even facilitating spread of more AbR in the long term. Apparently ‘simple’ interventions are frequently complex and unpredictable in their effects (Read et al. 2011; Derde et al. 2014; McVicker et al. 2014; Nicolle 2014). This realization argues for the set-up of more fundamental research programmes as well as a broader education during training to create awareness among young trainees (Nesse et al. 2010; Ashbolt et al. 2013; Viana et al. 2014).

Complex interventions are also those that associate different interventions influencing various factors involved in a complex problem, as AbR. Because of the frequent feedback loops occurring in complex systems, it is difficult that a single intervention could be sufficient to significantly influence the long-term outcome of the target objective. On the contrary, associations of interventions might have a synergistic effect.

### On the possibility of effective interventions and on the need for them

The first key question, from a Public Health perspective, is what we can realistically expect from the application of the certainly complex interventions that might be required for reducing the risks associated with AbR. It has been suggested that measures that might be successful in early stages of the development of resistance or in hospitals or countries with low rates of AbR have small or no value in geographical areas where resistance is already an established biological phenomenon (Baquero et al. 2011). Considering the current level of ‘resistance occupancy’ in many environments, increasingly linked in a globalized world, it will be extremely difficult in the medium term to be excluded from the invasion of AbR. Beyond the red line, resistance will invade and reinvade free environments from their global reservoirs, and therefore, our best realistic hope could be only to partially contain or slow the flow of the resistance torrents.

The second key question, in a Public Health perspective, is whether we will really need of any intervention on AbR in the medium and long-term future. AbR is a medical and Public Health problem only because we are currently using drugs that can be inactivated by resistance mechanisms. Let us imagine a future in which the number of novel antimicrobial drugs could be multiplied several times. The threat of resistance to current drugs will be strongly devaluated, as it is chloramphenicol resistance in a medical landscape that has renounced (the reasons are not entirely clear) the use of this drug. On the other hand, an entirely new way of treating infections, for instance based on the modulation of pathogenic immune-inflammatory host's responses, being microbes only the occasion for such effect has recently been suggested (Ali et al. 2014). Preliminary observations indicate that noninferiority of therapy of ibuprofen of symptomatic uncomplicated UTI compared to ciprofloxacin (Bleidorn et al. 2010). In mild respiratory tract infections, ibuprofen anti-inflammatory treatment has already largely substituted antibiotics as ‘antifever’ therapy and seems to be more effective than antibiotic treatment to reduce cough, which is the most disturbing symptom for patients with noncomplicated acute bronchitis with purulent sputum (Llor et al. 2011)*,* and even in chronic lung infections such as that of cystic fibrosis (Konstan et al. 1995; Konstan 2008).

Using the precautionary principle, we feel obliged in our days to examine a number of interventions aimed at slowing down the evolution (emergence, invasion, occupation) of AbR. In this work, we are particularly focusing the interventions that might have a Public Health dimension, exerting a societal impact by acting on the eco-epidemiology of AbR rather than on the prevention of AbR in the individual patient.

## Identifying priorities for intervention

As can easily be deduced from the former sections, interventions on emergence should influence invasion, those acting on invasion influences also occupation, and interventions on occupation are determinant for emergence. Because of that, we listed below general interventions that might reduce the entire loop of processes shaping AbR. Some of them are already implemented in our days, and some others should be considered for future application.

### Interventions to reduce the absolute number of AbR organisms

First, interventions to reduce *selection* of resistant organisms include the following: (i) Overall reduction of antibiotic use in humans, animals and agriculture; early diagnosis of bacterial and viral diseases and infection severity will benefit the identification of situations deserving antibiotic therapy (http://ec.europa.eu/health/antimicrobial_resistance/policy/index_en.htm). (ii) Substitution of antibiotics by other drugs (such as anti-inflammatory compounds) in mild infectious diseases (Konstan et al. 1995; Melino et al. 2014). (iii) Promotion of antibiotic combinations, sequential use, cycling strategies or mixing strategies of different drugs (Perron et al. 2012a; Baquero et al. 2014) that have demonstrated positive effects on the reduction of antimicrobial resistance, and phage–antibiotic combinations in treating initially sensitive bacteria (Zhang and Buckling 2012). (iv) Reduction in the local selective effect of antibiotics, as with inactivating or antibiotic-adsorbing (‘sponge’) compounds (Grall et al. 2013). (v) Appropriate doses, generally high, to surpass *mutant prevention concentration* and to avoid *mutant selection window* (Zhao and Drlica 2002; Ankomah and Levin 2014; Vasseur et al. 2014). (vi) Patients’ and general public educational interventions to reduce self-prescription and demands for antibiotics to prescribers, based on the ‘principle of fear’, that is, there is an increase in the individual risk of morbidity and mortality of infectious diseases associated with personal exposure to these drugs (as it occurs for tobacco, heart diseases and lung cancer) (Baquero 2007; Charani et al. 2014).

Second, interventions to reduce *host colonization* by resistant organisms include the following: (i) Vaccination-based interventions to decontaminate (mucosal immunity) colonized hosts; indeed, antipneumococcal and anti-*Haemophilus* vaccination have significantly decreased AbR rates in these organisms, and there is active vaccination research focused on *Staphylococcus aureus* and *Escherichia coli* (Kyaw et al. 2006; Brumbaugh and Mobley 2012; Henriques-Normark and Normark 2014). Viral vaccines may indirectly have effects against AbR bacteria (Patel et al. 2004); http://www.immunizationinfo.org/es/issues/general/vaccines-and-antibiotic-resistant-bacteria). (ii) Probiotic–prebiotics, cation-feed supplements in farms, clonal and microbiota-transplantation procedures in the aim of ecological displacement of resistant populations can have disparate effects (Bednorz et al. 2013; Ubeda et al. 2013; Macfarlane 2014; Pamer 2014). (iii) Drug-based decontamination interventions with nonabsorbable antimicrobials without cross-resistance with therapeutic antibiotics. (Daneman et al. 2013). (iv) Interventions to reduce conditions enlarging the colonization with gamma-Proteobacteria and Firmicutes, the taxa more frequently involved in AbR, in the human and animal microbiota, including modifications to reduce high-fat diet.

Third, interventions to reduce *environmental colonization* by AbR organisms. (i) Decontamination of human and animal wastewater and sewage in hospitals, farms, but also urban sewage, treatment plant discharge, sewage overflow, run-off manure-fertilized agricultural fields and livestock farms; particular interest of decontamination in developing countries where the resistant organisms released in the environment have larger chances to be reacquired by humans and animals. (ii) Surface microbial decontamination of floors and equipment in hospitals and farms. (iii) Prevention of environmental releases and decontamination of antimicrobial substances, including biocides, metals and industrial pollutants, as influencing the environmental selection of resistant organisms. (iv) Bioremediation of contaminated compartments with the substitution of resistant bacteria with susceptible ones, eventually with/or specific bacteriophages. This has also been suggested at patient level with faecal transplant with susceptible bacteria in colonized patients with highly resistant bacteria (Baquero et al. 2008; Wellington et al. 2013).

### Interventions to reduce the transmission of AbR organisms

Firstly, interventions to reduce *host-to-host transmission* of AbR organisms the following: (i) Health workers hand washing in hospitals and long-term care facilities, with particular attention of the possible ‘best transmitters’ (Centers for Disease Control and Prevention 2013; Del Campo et al. 2014). (ii) Containment measures for patients colonized with AbR organisms, and, ideally, on any individual under antibiotic therapy. (iii) Vaccination against viral diseases helping spread bacterial organisms. (iv) Prevention of human and animal crowding. (v) Early identification of colonized patients by resistant bacteria using rapid diagnostics tools prior to admittance in risk areas for transmission of AbR bacteria (e.g. intensive care units) (Smolinski et al. 2003; Derde et al. 2014; Moore et al. 2014; Tomson and Vlad 2014).

Secondly, interventions to reduce *water and food to host transmission* of antibiotic-resistant bacteria include the following: (i) Drinking, washing and bathing water decontamination, particularly in nondeveloped areas; methods range from conventional chlorination to the use of phages in contaminated surface water streams, (ii) prevention of meat contamination by intestinal bacteria in slaughterhouses, (iii) hygienic procedures in food handlers, (iv) safe food storage to prevent growth of antibiotic-resistant bacteria, (v) use of phages to eliminate dangerous high-risk clones from food and eventually inhibiting emerging outbreaks (Maura and Debarbieux 2011; Merabishvili et al. 2012), (vi) irradiation of water and food (Smolinski et al. 2003; Ashbolt et al. 2013; Wellington et al. 2013; Tomson and Vlad 2014; Viana et al. 2014).

### Interventions aimed at the maintenance and biorestoration of susceptible populations

Interventions to fight against AbR should go beyond the control of AbR organisms. AbR evolves in a multilevel space of independent units of selection, encompassing genes, integrons, transposons and integrative-conjugative elements, plasmids and chromosomal regions (Baquero 2004; Baquero et al. 2013). Each one of these evolutionary units is a potential target for interventions including the application of novel eco-evo ‘drugs’ (Baquero et al. 2011). In AbR, the aim of biorestoration is the recovery or reimplantation of the original susceptible populations in particular environments, substituting the resistant ones. We should help normal susceptible bacteria to ‘win the battle’ against resistant ones. Recent advances in synthetic biology might influence the advances in this field in the future. As in the former paragraphs, these interventions should influence the absolute number and the transmission abilities of these units. A number of these possible interventions are enumerated below (revised in Baquero et al. 2011).

First, interventions aimed at *selecting susceptible populations* over resistant ones include the following: (i) Enhancers of the biological cost of resistance, so that the growth rate of resistant organisms decreases over that of susceptible ones. (ii) Clonal or population replacement of resistant organisms by homogenic susceptible ones, eventually using specific selection with nontherapeutic antimicrobials. (iii) Antagonistic pleiotropy strategies, so that resistance to a given antibiotic provokes susceptibility to another whose susceptibility should be preserved (Perron et al. 2007; Brumbaugh and Mobley 2012; Van den Abbeele et al. 2013; Henriques-Normark and Normark 2014).

Second, interventions to promote *early establishment of susceptible microbiota* in human and animal newborns, interrupting the ‘vertical transmission of resistance’ (Van den Abbeele et al. 2013).

Third, interventions to decrease *horizontal gene transfer of resistance genes* to susceptible populations include the following: (i) Application of ‘drugs’ preventing plasmid (and ICE) stability and conjugation, particularly from the recipient cells, as unsaturated fatty acid derivatives and tanzawaic acids, or antipheromone compounds. (ii) Application of wide host range cocktails of plasmid-dependent phages directed to plasmid-encoded sex apparatus (Baquero et al. 2011; Jalasvuori et al. 2011; Ojala et al. 2013).

Four, interventions aiming at specific resistance gene decontamination in bacterial populations include the following: (i) Use of antisense oligomers, and external guide sequence antisense oligoribonucleotides (Soler Bistué et al. 2009; Sala et al. 2012). (ii) Utilization of the CRISPR/Cas bacterial immune system to disrupt resistance genes (Garneau et al. 2010).

## The strategies to use interventions: from theory to feasibility

In the above sections, we have examined the difficulties and also some of the current or expected possibilities of interventions to fight AbR. Most of these interventions are based on the control of the complex evolutionary processes shaping at multiple levels the emergence, invasion and occupation of the environments by AbR organisms. Intervention to control and repair complex systems is hard work as we can no longer rely on classic reductionistic approaches. In fact, widespread current ‘strategies’ to fight against AbR have proved to have a limited (local) success and most probably are insufficient to deal with the multidimensionality of the problem (Baquero et al. 2011). The discovery of novel antibiotics is indeed a major condition to successfully treat patients infected with resistant organisms, but the cumulative history of chemotherapy indicates that by themselves novel antibiotics do not solve the problem of AbR. We should face complexity by applying tools of complexity, expanding conventional understanding of scientific representation of the problem of AbR to reach a more solid platform for decision-making. Of course, decision-making should consider feasibility. In Table 1, we have summarized the feasibility of the interventions that we have proposed throughout this review versus the impact they might have on AbR, considered as a global problem. It is important to note the term ‘feasibility’ means ‘current feasibility’, but not necessarily lack of feasibility in the future. That is why low-feasible interventions today should be considered.

**Table 1:** Feasibility–impact matrix of interventions against antimicrobial resistance

	Impact		
Feasibility	High	Medium	Low
High	N – Substitution of antibiotics by other drugs (as anti-inflammatory compounds) in mild infectious diseases	N – Antiviral vaccination, reducing use of antibiotics in viral diseases	
	N – Vaccination-based interventions to decontaminate colonized hosts with resistant bacterial clones		
Medium	N – Reduction in the local selective effect of antibiotics, as with inactivating or antibiotic-adsorbing compounds	N – Patient's and general public educational interventions to reduce self-prescription	N – Appropriate doses, generally high, to mutant prevention concentration
	N – Decontamination of human and animal wastewater and sewage in villages, hospitals and farms	N – Overall reduction of antibiotic use in humans, animals and agriculture	N – Prevention of environmental releases and decontamination of antimicrobial substances, including biocides, metals and industrial pollutants
	T – Early identification of colonized patients by resistant bacteria prior to admittance in risk areas for transmission of resistant bacteria	N – New antimicrobial agents, antibiotic combinations, sequential use, cycling or mixing strategies of different drugs	
	T – Interventions to reduce water and food to host transmission of antibiotic-resistant bacteria	T – Interventions to reduce host-to-host transmission of antibiotic-resistant organisms in health workers (including hands washing) and farms	
	T – Decontamination procedures in cooking and handling of raw food	N – Surface microbial decontamination of floors and equipment in hospitals and farms	
		E – Early establishment of susceptible microbiota in human and animal newborns	
Low	E – Interventions aimed to select susceptible populations over resistant ones	T – Prevention of human and animal crowding	N – Probiotic-prebiotics, clonal and microbiota-transplantation procedures for ecological displacement of R bacteria
		T – Containment measures for patients colonized with antibiotic resistance organisms	
	E – Clonal or population replacement of resistant organisms by homogenic susceptible ones	T – Prevention of meat contamination by intestinal bacteria in slaughterhouses	N – Interventions to reduce conditions enlarging the colonization with gamma-Proteobacteria and Firmicutes
	E – Drug-based decontamination interventions for R bacteria with nonabsorbable antimicrobials	T – Irradiation of water and food	
		E – Antagonistic pleiotropy strategies for selecting susceptible populations	
	E – Interventions directed to the maintenance and biorestoration of susceptible organisms	E – Interventions to decrease horizontal gene transfer of resistance genes to susceptible populations	E – Interventions aiming to specific resistance gene decontamination in bacterial populations

N, interventions acting on number; T, interventions acting on transmission; E, interventions acting on ecology bioremediation of antibiotic resistance.

Most probably, an efficient way of applying these interventions is to use several of them simultaneously, as they are not simple solutions to complex problems. These combinations of interventions should be tailored to be applied to for their application to in specific circumstances, considering the different levels of risk for Public Health. As stated before, complex interventions in complex systems should be closely monitored to prevent unexpected outcomes. In most cases, they should be applied to solve specific problems (for instance at early stages of the detection of a novel resistance problem) and should encompass the infected patients, but also the patients’ group and their environment in an integrated way. It is also worth noting that many of our proposed interventions to control AbR are identical to those required to control infectious diseases, or even to decrease general health risks in the population. Consistently with the classic concepts of McKeown (1979), the advancements in general welfare and sanitation of human populations, particularly in nondeveloped countries, will result in a clear amelioration in the world rates of AbR, because resistance to antibiotics is only one of the many facets of Global Health.
